# GIS-based Visualization of Elemental Distribution in *Neoboletus Luridiformis* Fruiting Body

**DOI:** 10.1007/s12011-024-04320-3

**Published:** 2024-07-27

**Authors:** Július Árvay, Martin Hauptvogl, Lenka Demková, Ivona Jančo, Silvia Jakabová, Mirosław Mleczek

**Affiliations:** 1 Institute of Food Sciences, Faculty of Biotechnology and Food Sciences, Slovak University of Agriculture in Nitra, Tr. A. Hlinku 2, Nitra, 949 76 Slovak Republic; 2 Department of Sustainable Development, Faculty of European Studies and Regional Development, Slovak University of Agriculture in Nitra, Tr. A. Hlinku 2, 949 76 Nitra, Slovak Republic; 3 Department of Ecology, Faculty of Humanities and Natural Sciences, University of Prešov, 17. Novembra 1, Prešov, 081 16 Slovak Republic; 4 AgroBioTech Research Center, Slovak University of Agriculture in Nitra, Tr. A. Hlinku 2, Nitra, 949 76 Slovak Republic; 5 Department of Chemistry, Poznań University of Life Sciences, Wojska Polskiego 75, Poznań, 60– 625, Poland

**Keywords:** Mushroom, Bioconcentration, Element, Distribution, *Neoboletus Luridiformis*, GIS

## Abstract

The fruiting body of *Neoboletus luridiformis* (Scarletina bolete) mushroom was used to determine the level of bioconcentration and subsequent distribution of seventeen elements (Ag, Al, Ba, Ca, Cd, Cr, Cu, Fe, K, Mg, Mn, Na, Ni, Pb, Se, Sr, and Zn). A two-centimeter-thick vertical section of the entire fruit body was divided into 101 partial sub-samples where the contents of the studied elements were determined using ICP OES. The actual distribution of the elements in the fruiting body profile was visualized using a GIS interpolation method resulting in distribution maps. The study provides valuable insights into the distribution patterns of 17 elements within the fruiting body of *N. luridiformis*. Based on the visualization of the elemental content, the determined elements can be divided into three categories. Elements accumulated primarily *(i)* in the cap (Al, Ag, Ca, Cd, Cu, Fe, K, Mg, Ni, and Zn), *(ii)* in the stipe (Ba, Mn, Na, Pb, and Se), and *(iii)* elements with non-specific distribution (Cr and Sr). Since such detailed information supported by graphical visualization has not been published to date, the information in this study will help to better understand the accumulation and distribution of elements within the fruiting bodies of wild as well as cultivated mushroom species.

## Introduction

 Mushrooms can absorb high amounts of elements (biogenic and toxic) from the substrate through their mycelium, even if the concentrations of elements in the substrate are low, and then transport them to the aboveground parts during fructification [1]. It is confirmed by bioconcentration characteristics, which are higher in mushrooms than in vascular plants [2, 3]. Due to the ability to toxic elements bioaccumulation, mushrooms (especially from contaminated areas) may pose a risk to human health when consumed in excess [4]. The content of toxic (and other) elements in mushrooms’ fruiting bodies depends on geogenic and anthropogenic sources [5].

In recent decades, a large number of articles have addressed the issue elemental composition and their bioconcentration in various species of macroscopic (wild and cultivated) mushrooms. The uptake and subsequent distribution of elements during fructification into the fruiting body are affected by several factors (chemical composition of the substrate, location, species of mushroom, climatic conditions, age of the mycelium, number of fructification cycles, speed of fructification, or age of the fruiting body, etc.). However, there are still many uncertainties about these factors and more research is needed to shed light on this issue. The majority of studies focused on the elemental distribution in the cap and stipe [5–13]. One study divides the fruiting body into several anatomical parts, such as cuticle, flesh of cap, tubes and pores and stipe [3]. Few studies discuss elemental transport into the fruiting body and within it in more detail [14–16], noting that most elements are distributed unevenly within the fruiting body. These studies, however, used highly sophisticated and financially demanding analytical methods and instruments, such as laser ablation coupled to ICP. In our previous work [17], we addressed the mapping of Hg distribution within the fruiting body of *N. luridiformis* using the current basic analytical equipment of a chemical laboratory and freely available QGIS software resulting in a detailed heatmap of the Hg content in the fruiting body.

The aim of this study was to evaluate the usefulness of the GIS approach to the detailed analysis of the concentration and distribution of 17 elements within the fruiting body of *N. luridiformis*. The species was choosen as it belongs to the often collected and consumed *Boletaceae* family. It is also a species that is among the frequently occurring species, and the fruiting body is characterized by integrity. This work brings an important knowledge on the behavior of chemical elements in the process of fructification and their distribution within the forming fruiting body, where the elements are translocated from the mycelium. As it is presented in the work, some toxic and potentially toxic elements accumulate in different anatomical parts. The distribution within the cap and stipe has been known for a long time, but graphic imaging methods are able to define in more detail the individual zones within the fruiting body where there is a higher (or lower) accumulation of chemical elements. The graphic representation of the distribution of concentrations of individual elements may be serve for further research on this issue. Our hypothesis was that the GIS approach would be easier and cheaper alternative to the laser ablation method providing very similar results.

## Materials and Methods

The one fully matured fruiting body of *Neoboletus luridiformis* (Rostk.) Gelardi, Simonini & Vizzini 2014 was sampled and prepared as described in detail in our previous paper [17] that also includes the basic characteristics of *Neoboletus luridiformis* (Rostk.) and the sampling location. We chose this species due to its belonging to the *Boletaceae* family, which includes a wide group of commonly collected and consumed species. The representatives of the family are known for their relatively high bioaccumulation properties and tubular hymenophore.

**Fig. 1:**
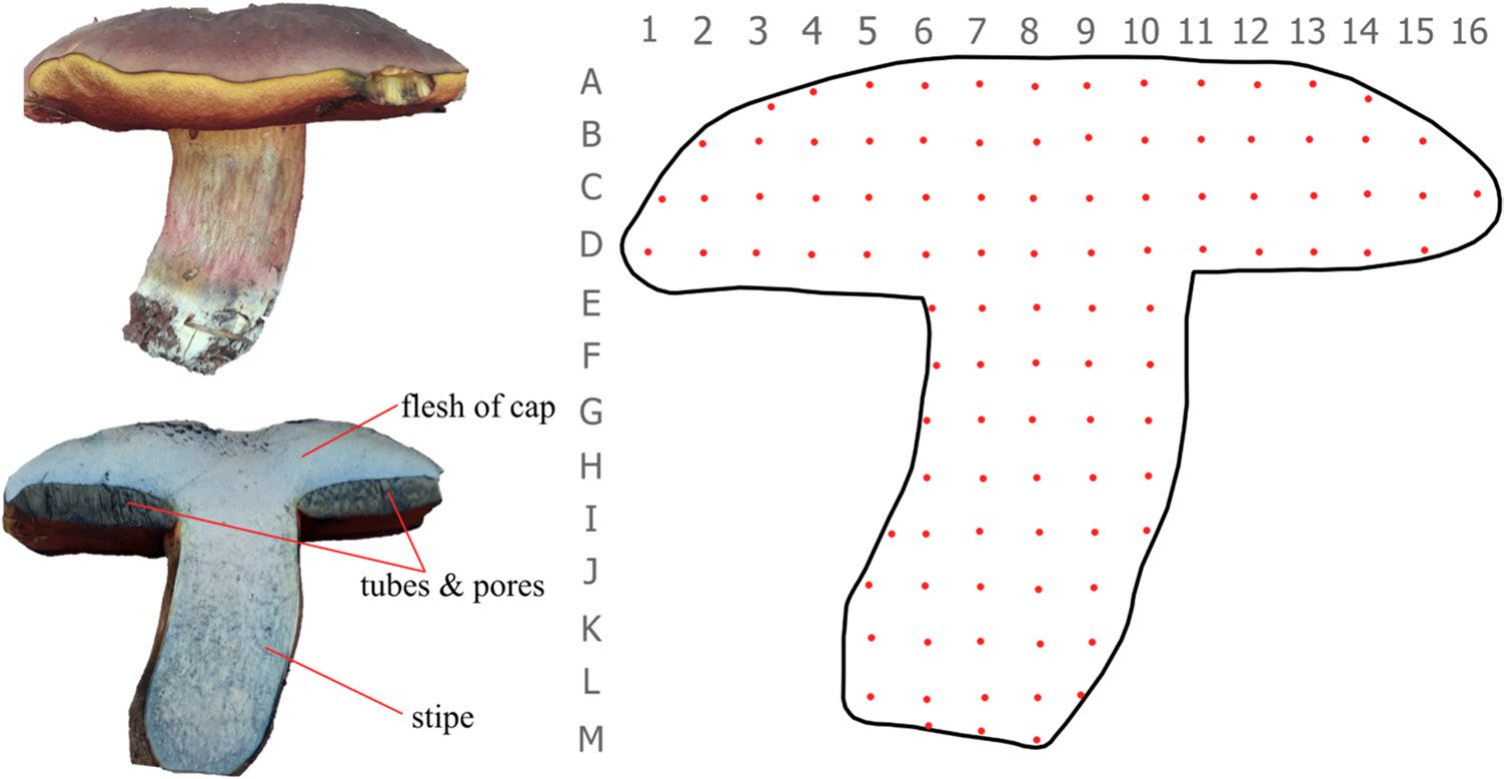
The *N. luridiformis* fruiting body cut to determine the spatial distribution of elements with the sampling points (raster 1 × 1 cm)

In total, 17 elements divided into 4 separate groups of elements: Major Essential Elements – MEEs (Ca, K, Mg, and Na), Essential Trace Elements – ETEs (Cr, Cu, Fe, Mn, Ni, Se, and Zn), Trace Elements with Detrimental Health Effect – TEWDHE (Ag, Ba, Cd, Pb) and Nutritionally Nonessential Elements – NNEs (Al and Sr) determined by ICP OES. The detailed preparation of the 101 partial samples of the fruiting body is presented in Fig. 1.

Concentrated (67%) nitric acid (Honeywell, Saint-Germain-en-Laye, France) and 30% hydrogen peroxide (Supelco Inc., Bellefonte, Pennsylvania, USA) of trace purity were used to analyze the content of the studied elements in mushrooms. Approximately 150 mg of a dried and homogenized partial sample was weighed to four decimal places on analytical balances KERN ABT 120-5DM (KERN & Sohn GmbH, Balinger, Germany) into PTFE digestion tubes, where 8 mL of HNO_3_ and 2 mL of H_2_O_2_ were added. The samples were then prepared in a closed microwave digestion system ETHOS-One (Milestone, s.r.l., Sorisole BG, Italy). The process of digestion was carried out according to the following program: (I) 0–15 min.: increase to 200 °C (pressure increase to 40 Bar); (II) 15–30 min.: temperature fixed at 200 °C (40 Bar, 1.8 kW); (III) 30–50 min.: Cooling. After microwave digestion, the samples were filtered through qualitative filter paper, Grade 595 with a porosity of 4–7 μm (Whatman, Maidstone, UK) into volumetric flasks and filled up to the final volume of 50 mL with deionized water using a LabAqua HPLC device (Biosan, Riga, Latvia). The analysis of the elemental content and quality assurance were carried out on the instrument Agilent ICP OES 720 (Agilent Technologies Inc., Santa Clara, CA, USA) coupled with the autosampler SPS 3 (Agilent Technologies Ltd., Malaysia) according to Shah et al. [18] and shown in the Table 1. All determined element contents in the text are expressed as mg/kg on dry weight basis (DW).

**Table 1:** ICP OES determination parameters

Elements	Wavelength [nm]	LoD [µg/L]	LoQ [µg/L]	*R*
**Ag**	328.068	0.30	0.99	0.999913
**Al**	167.019	0.20	0.66	0.999749
**Ba**	455.403	0.03	0.10	0.999713
**Ca**	315.887	0.01	0.03	0.999810
**Cd**	226.502	0.05	0.17	0.999938
**Cr**	267.716	0.15	0.50	0.999908
**Cu**	324.754	0.30	0.99	0.999941
**Fe**	234.350	0.09	0.33	0.999702
**K**	766.491	0.30	0.99	0.999411
**Mg**	383.829	0.01	0.03	0.999836
**Mn**	257.610	0.03	0.10	0.999784
**Na**	589.592	0.15	0.50	0.999343
**Ni**	231.604	0.30	0.99	0.999816
**Pb**	220.353	0.80	2.64	0.999850
**Se**	196.026	2.00	6.61	0.999974
**Sr**	407.771	0.01	0.03	0.999490
**Zn**	206.200	0.20	0.67	0.999881

***LoD***: limits of detection; ***LoQ***: limit of quantification; ***R***: correlation coefficient of calibration curves

### Data Processing and Interpolation

The data on element contents were processed and visualized in the open-source QGIS software. The values representing contents of 17 elements in individual partial samples were interpolated using the B-spline interpolation method [17]. Interpolation is a commonly used GIS technique to create a continuous surface from discrete points. The B-spline function is applied to the multivariate data. In this case, B-spline is performing a bilinear spline interpolation with Tykhonov regularization, with the output being a 2D vector points map. From a theoretical perspective, the interpolating procedure takes place in two parts: the first is an estimate of the linear coefficients of a spline function derived from the observation points using a least squares regression; the second is the computation of the interpolated surface (or interpolated vector points) [19]. As used here, the splines are 2D piece-wise non-zero polynomial functions calculated within a limited, 2D area. B-splines are considered a powerful tool commonly used in statistics to model smooth functions [20].

## Results and Discussion

### Major Essential Elements – MEEs

**Fig. 2:**
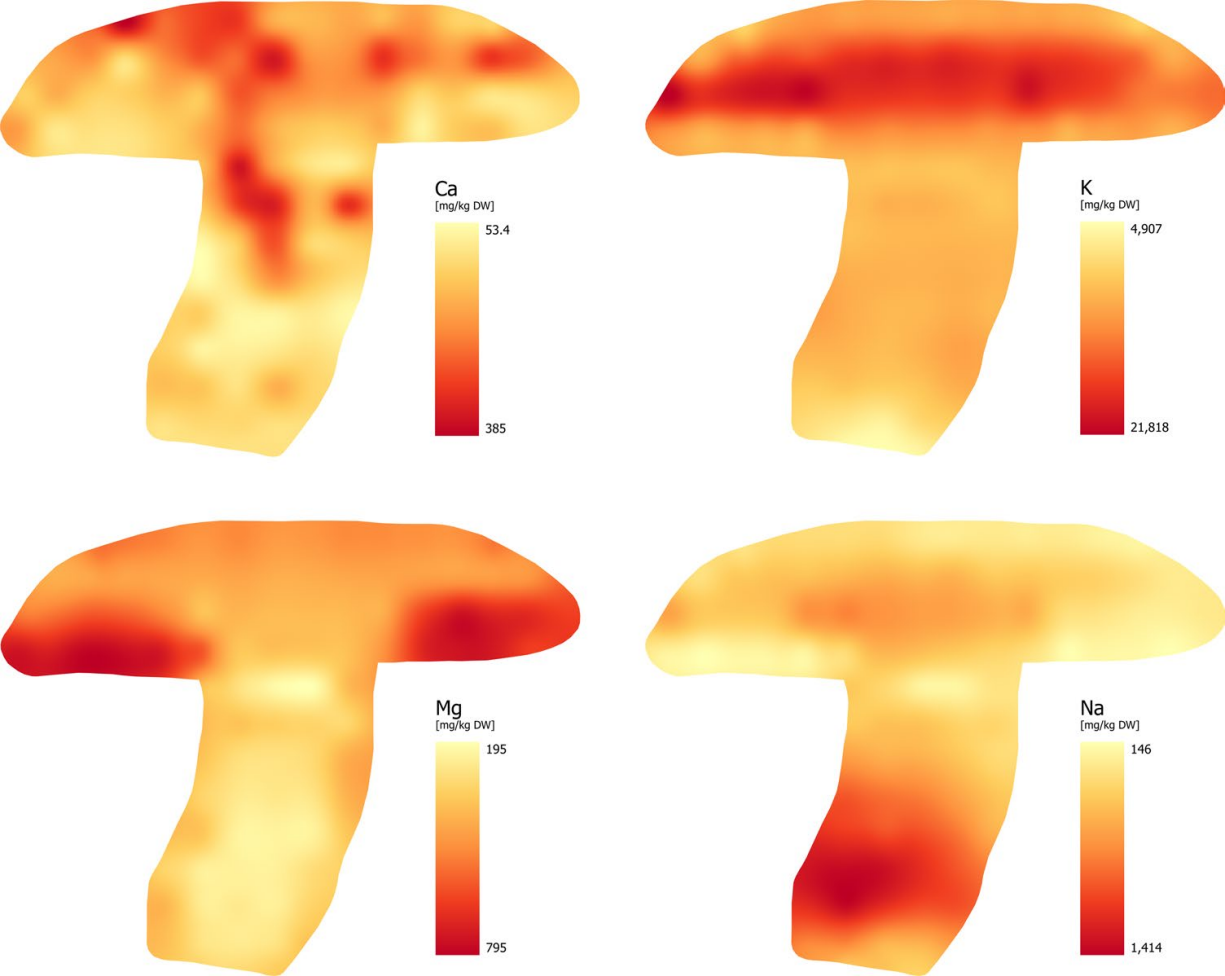
Distribution of Major Essential Elements – MEEs (Ca, K, Mg and Na)

Based on the typical elemental composition of mushrooms, there are 7 elements (Ca, K, Mg, Na, P, S, and Cl) whose content in the dry matter typically ranges from 500 mg/kg and more [21]. The study focused on tracking the content and distribution of the first four elements (Ca, K, Mg, and Na). Their distribution maps are shown in Fig. 2.

### Calcium (Ca)

Dimitrijević et al. [22] reported that Ca is found in significantly lower concentrations in the fruiting bodies of edible mushrooms than K or P. The Ca content in members of the *Boletaceae* family ranges from 100 to 300 mg/kg, but it can be higher in some species (more than 2,000 mg/kg). However, according to Falandysz et al. [23], the Ca content in the fruiting body of *B. edulis* ranged from 5.60 to 420 mg/kg. Kalač [24] found that in studies conducted after 2010, the Ca content in mushrooms was most reported to be between 50.0 and 750 mg/kg. Calcium is not absorbed and accumulated by mushrooms to a significant degree (bioconcentration factor: 0.68 to 4.90) and its content in mushrooms is generally lower than in vegetables. The resulting Ca concentration ranged from 53.5 to 385 mg/kg of the fruiting body. Visualization revealed that the highest concentrations of Ca were concentrated in the cap, particularly in lines A – C, similar to the distribution of K. However, the distribution of Ca is uneven in these parts of the cap. The distribution of Ca was as follows: flesh of cap > tubes and pores > stipe.

### Potassium (K)

Potassium is usually an abundant mineral element in various mushroom species making them a relevant source of this element [25, 26]. The bioaccumulation potential of wild and cultivated mushrooms varies and depends on the particular mushroom species and the K concentration in the substrate. However, the bioaccumulation potential of mushrooms for this element is high. The level of K uptake is typically in the range of units to hundreds of times its concentration in the underlying substrate (depending on the species) and in extreme cases up to thousands, which is confirmed by the findings of many authors [27–33]. Its standard concentration in the entire fruiting body ranges from 1.0 to 3.5% (10,000 to 35,000 mg/kg), with almost 70% of mushroom species (from 400 studied) accumulating potassium in the range of 20,000 to 50,000 mg/kg [34]. In the case of cultivated mushrooms, the contents of macroelements are generally lower [24]. Potassium was not evenly distributed within the fruiting body, and its concentration in different anatomical parts decreased in the following order: flesh of cap > tubes and pores > stipe. The K concentration in the entire fruiting body ranged from 4,910 to 21,800 mg/kg. Overall, the K concentration was higher than other elements. Based on our findings, the highest K concentration is localized in the cap above the hymenophore and compared to Ca, its distribution within this part is homogeneous (Fig. 2).

### Magnesium (Mg)

According to Seeger and Becket [35], the average Mg content in most macroscopic fungi ranges from 0.08 to 0.18% (800 to 1,800 mg/kg). Among the 402 species studied, the *Coprinaceae* family had the highest content while the *Boletaceae* family was characterized by the lowest values.

In our study, the Mg content in the fruiting body ranged from 195 to 794 mg/kg. In general, the distribution of Mg in fruiting bodies is as follows: hymenophore > stipe = flesh of cap = spores [24]. Our findings confirm that Mg content was highest in the hymenophore (tubes and pores > flesh of cap ≥ stipe) where it was evenly distributed. This suggests that Mg is one of the elements essential for the spore formation.

### Sodium (Na)

Among essential major elements, Na is unique in its distribution within the *N. luridiformis* fruiting body. The stipe, particularly its lower part, exhibits the highest bioconcentration capacity (Fig. 2). Interestingly, the hymenophore (tubes and pores) has the lowest Na concentration. A comprehensive study by Seeger et al. [36] examined Na content in 465 mushroom species. They found that the content mostly ranged from 100 to 400 mg/kg, with *Agaricaceae* family species exhibiting the highest bioconcentration potential, reaching an average Na content of 3,470 mg/kg.

In our study, the Na content varied between 146 and 1,410 mg/kg. This is a relatively wide range, but the intra-fruiting body distribution shows that the highest Na concentrations are localized in the stipe, specifically in lines H – L (617–1,410 mg/kg), and the lowest in the hymenophore part of the cap (line D) and the upper part of the stipe (line E). Available information on Na content in mushroom fruiting bodies presents conflicting findings on distribution. For example, Falandysz et al. [23] reported a significantly higher Na content in the stipe than in the cap of *B. edulis*, while Wang et al. [33] found the Na content in the cap and stipe to be almost identical in various *Boletaceae* species. The Na distribution in our study demonstrates the increased accumulation of this element in the stipe (stipe ≥ flesh of cap = tubes and pores).

### Essential Trace Elements – ETEs

This group includes Cr, Cu, Fe, Mn, Ni, Se and Zn. They are essential for living organisms, playing specific roles in biochemical processes. Chromium stands out, as only its trivalent form exhibits the essentiality, while the hexavalent form is harmful [37]. Typically, mushrooms contain these elements in amounts ranging from mere units to hundreds, possibly even thousands of milligrams per kilogram of dry matter in exceptional cases. However, when mushrooms originate from areas with increased levels of these elements due to geological or human-caused anomalies, their concentrations can become extreme, potentially leading to risks associated with mushroom consumption.

### Chromium (Cr)

Chromium is an essential trace element that exists in two oxidation states, Cr^3+^ and Cr^6+^. The hexavalent form is the one that poses a health risk [38]. The commonly reported Cr content in mushroom fruiting bodies is 0.5–10 mg/kg [6, 24].

There are discrepancies in the literature regarding the intra-fruiting body distribution of Cr. Dong et al. [39] reported that the Cr distribution in mushroom fruiting bodies is uneven, with higher concentrations in the stipes than in the caps (Cr ratio of 1.11 vs. 0.92 mg/kg in favour of the stipe). However, Kalač [24] reported that Cr is evenly distributed in the fruiting body.

In terms of Cr bioconcentration by mushrooms, Cr is not taken up by mushrooms in excessive amounts. In most published studies, the bioconcentration factor is below 0.50, with slightly higher values being characteristic of the stipe compared to the cap [31, 40]. In general, the health risk to consumers from Cr content is low, however, mushrooms take up hexavalent Cr to a greater extent than trivalent Cr [38].

**Fig. 3:**
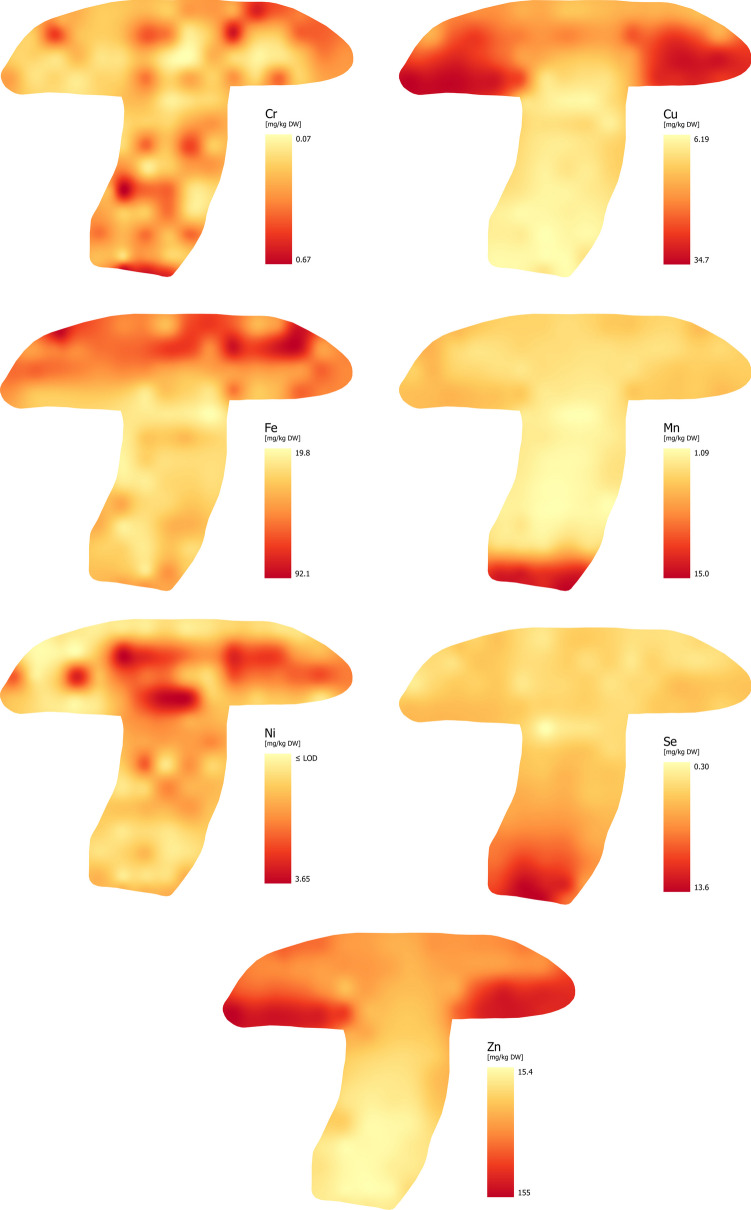
Distribution of Essential Trace Elements –ETEs (Cr, Cu, Fe, Mn, Ni, Se and Zn)

Based on the results (Fig. 3), it can be concluded that the distribution of Cr in the fruiting body was relatively homogeneous (except for some subsamples). The Cr concentration in the whole fruiting body ranged from 0.07 to 0.67 mg/kg, with a standard deviation of 0.29 mg/kg. The distribution of Cr was as follows: tubes and pores = flesh of cap = stipe.

### Copper (Cu)

The Cu content in wild mushrooms usually ranges from < 10 to 75 mg/kg, while in cultivated mushrooms it is about half that of wild mushrooms [24]. Of course, substantially higher concentrations can be found in contaminated areas [41–43]. The species that accumulate the most Cu include *X. badius* and *M. procera*, but species of the family *Boletaceae* also have a non-negligible bioconcentration capacity [44]. Information on the distribution of Cu within the fruiting body is conflicting, but most authors agree that higher concentrations of Cu are found in the cap than in the stipe [6, 33].

Our results support the findings of most authors that Cu is primarily bioconcentrated in the hymenophore (Fig. 3). The Cu content in the whole fruiting body ranged between 6.19 and 34.6 mg/kg, while in the cap it was 10.2–34.6 mg/kg and in the stipe 6.19–12.0 mg/kg. The highest concentrations (13.8–34.6 mg/kg) were in lines C and D (hymenophore). The higher bioconcentration capacity of the hymenophore is related to the higher biological activity of this part, which is probably caused by the higher content of metal-binding compounds [45]. The distribution of Cu was as follows: tubes and pores > flesh of cap > stipe.

### Iron (Fe)

The Fe content in wild mushrooms is relatively wide (< 50–1,000 mg/kg), with some species having higher content (*Boletaceae* family). The Fe content in cultivated species is lower (< 50–300 mg/kg) [46]. Despite the high Fe concentrations, mushrooms are generally considered to be Fe bioexcluders, as the typical BCF values for this element are generally 0.1 and below [31, 42]. Very high concentrations of Fe (4,660 mg/kg) were recorded in *B. edulis* [33]. The Fe distribution within the fruiting body of higher fungi is diverse. In general, Fe is evenly distributed throughout the fruiting body, but its content is higher in the cap than in the stipe in some species [24]. It is also true that higher concentrations of Fe are found in the cap peel [47].

The Fe concentration in the *N. luridiformis* ranged from 19.8 to 92.1 mg/kg. Figure 3 shows that the highest Fe concentrations are in the middle and upper part of the cap (lines A – D), where the range of values for the content of this element is between 27.5 and 92.1 mg/kg. The average concentration was 58.0 mg/kg. When comparing the three basic parts of the fruiting body, the Fe content was as follows: flesh of cap ≥ tubes and pores > stipe.

### Manganese (Mn)

The commonly reported Mn content ranges from < 25.0 to 75.0 mg/kg in wild mushrooms and the Mn content in cultivated mushrooms is < 25.0 mg/kg [24]. Some species, such as *B. edulis* or *M. procera*, commonly exhibit concentrations above 100 mg/kg [48]. Similar to Fe, the distribution of Mn within the fruiting body depends on the species, with most edible species accumulating this element in the stipe [49]. In some cases, the distribution is even [50]. The Mn bioconcentration potential of mushrooms is very low or non-existent and typically is less than 0.50 (exceptionally 1.00) [31, 42]. This suggests that mushrooms are bioexcluders of Mn.

Our findings support the claim that Mn is primarily accumulated in the stipe. The Mn content in the whole fruiting body ranged from 1.08 to 15.0 mg/kg, with the highest concentration recorded in lines L and M (Fig. 3). Regarding the three basic anatomical parts, the Mn accumulation was as follows: stipe > flesh of cap = tubes and pores.

### Nickel (Ni)

Based on available data, the typical Ni content in mushrooms ranges from 0.50 to 5.00 mg/kg [24, 51]. In some cases, where the Ni content was also high in the soil, or the mushrooms came from polluted areas, the Ni content may reach > 10.0 mg/kg [52]. The distribution of Ni in the fruiting body is largely dependent on the species, and it is not possible to definitively define its bioconcentration behavior. Similarly, it is also difficult to define its bioconcentration characteristics. Some studies define BCF for Ni as < 1 [49], while others define it as > 1 [31]. This suggests that Ni is rather not accumulated by mushrooms.

Based on our results, the Ni content in the whole fruiting body ranged from ≤ LOD to 3.65 mg/kg. The highest concentrations were recorded in the cap in lines B – D (Fig. 3). While Ni concentrated mainly in the cap, some areas of the stipe showed similar levels. This suggests that the distribution of Ni is rather uneven in the whole fruiting body, or species-dependent. The Ni concentration in the three basic anatomical parts was as follows: flesh of cap > tubes and pores = stipe.

### Selenium (Se)

The typical Se concentration in wild mushrooms ranges from < 0.50 to 5.00 mg/kg, with higher levels of this element not uncommon [24]. Its content is largely dependent on the species. The family *Boletaceae* has higher Se content by an order of magnitude than other species [23]. Data on the distribution of Se within the fruiting body vary. Some studies suggest that it is even [53], while other suggest that the Se content is higher in the cap than in the stipe (mainly in the hymenophore) [23].

In our study, the intra-fruiting body distribution of Se was different. Figure 3 shows that the highest concentrations of Se (6.37–13.6 mg/kg) were recorded in the lower part of the stipe (lines K – M). The Se concentration in the entire fruiting body ranged from 0.30 to 13.6 mg/kg.

There is very little research-based information on higher Se content in the stipe compared to the rest of the fruiting body. One study showed a higher concentration of Se in the stipe than in the cap of *A. bisporus* fortified with selenium [54]. However, it was a model experiment. The distribution of Se was as follows: stipe > tubes and pores ≥ flesh of cap.

### Zinc (Zn)

The commonly reported Zn content in both wild and cultivated mushrooms ranges from 25.0 to 125 (200) mg/kg, however higher contents are not uncommon [24]. Zinc is one of the most studied elements (similar to Cd, Cu, Hg, Pb) and for this reason there is a large amount of information available. Studies report the highest Zn concentrations in the cap, especially in the hymenophore [44, 55, 56], or in the peel of caps [47]. Mushrooms can be considered good bioaccumulators of Zn (BCF > 10), with the range of BCF values being quite wide, both between species and within species [31, 55].

The results showed that the Zn content in the whole fruiting body ranged from 15.4 to 155 mg/kg. The highest concentrations of Zn were found in the cap, especially in the hymenophore (lines C and D) (Fig. 3) ranging from 52.4 to 155 mg/kg. Our findings are fully consistent with generally accepted findings. The distribution of Zn was as follows: tubes and pores ≥ flesh of cap > stipe.

### Trace Elements with Detrimental Health Effect– TEWDHE

The studied species *N. luridiformis* belongs to the family *Boletaceae*, which also includes the King bolete (*Boletus edulis*). The latter species is characterized by a high tolerance for fungitoxic elements (Cd, Pb, Cu, etc.) due to its high prosperity on substrates with a high content of these elements [57], mainly in environmentally contaminated areas. This statement can be generalized to the whole family [22, 58]. This species is also characterized by a high bioconcentration capacity [59]. The combination of these two factors creates the assumption of an increased negative impact of consumption on health [60, 61].

### Silver (Ag)

In the available literature, which was summarized by Kalač [24], the most common concentrations of Ag in wild and cultivated mushrooms range from 0.5 to 2.0 mg/kg depending on a number of factors. Concentrations above 5.0 mg/kg can be found in mushrooms from contaminated areas. Based on the available data, macromycetes are effective accumulators of Ag [62, 63]. A significant factor affecting the Ag content in the fruiting body is the mode of nutrition. Borovička et al. [63] found that the average Ag content in mycorrhizal species was statistically lower than in saprotrophic species. In their study, the authors focused on a wide range of factors that could explain the accumulation of Ag in fruiting bodies. They state that almost all studies published up to that time had only dealt with Ag accumulation in the cap and stipe, but they considered even those data inconclusive. The hyperaccumulation ability of macrofungi to take up Ag is justified by possible biological importance, such as defense against natural enemies, protection against the negative effects of inorganic contaminants without proven positive effects (As, Cd and others), but also protection against microscopic fungi, bacteria, etc., which has not yet been clearly confirmed in mushrooms [64].

**Fig. 4:**
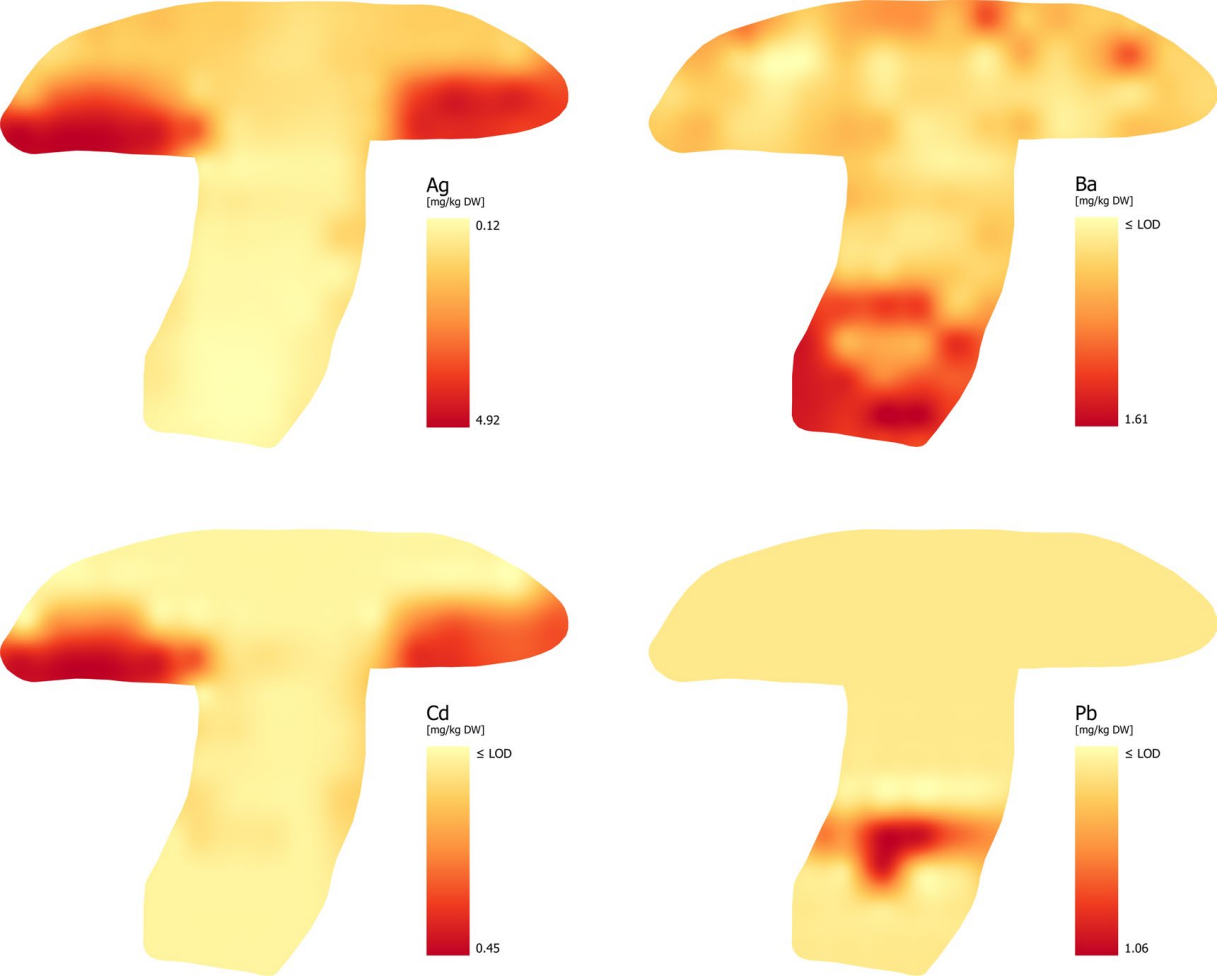
Distribution of Trace Elements with Detrimental Health Effect– TEWDHE (Ag, Ba, Cd and Pb)

Our results show that the tissue with the highest Ag content is the hymenophore (Fig. 4). The Ag concentration in the fruiting body ranged from 0.12 to 4.91 mg/kg (with an average concentration of 1.31 mg/kg), while in the tubes and pores (lines C and D) the Ag content ranged from 0.65 to 4.92 mg/kg (the average concentration was 2.69 mg/kg). The distribution of Ag was as follows: tubes and pores > flesh of cap > stipe.

### Barium (Ba)

Barium is a trace element whose content in mushroom fruiting bodies is around 2.0 mg/kg. Its concentrations above 10 mg/kg are typical for areas with specific geological conditions [6]. Unfortunately, data on the Ba concentration are limited. The available information suggests that its distribution between the stipe and cap is uniform [24] and bioconcentration characteristics suggest that mushrooms excrete Ba [65]. Our findings contradict the claim that the Ba distribution is uniform within the fruiting body (Fig. 4). The highest concentrations of Ba were recorded in the lower part of the stipe (lines I – M), ranging from 0.37 to 1.61 mg/kg. The Ba content in the whole fruiting body ranged from ≤ LOD to 1.61 mg/kg. The distribution of Ba was as follows: stipe > flesh of cap ≥ tubes and pores.

### Cadmium (Cd)

Cadmium is generally considered one of the most hazardous inorganic contaminants with very high persistence in all components of the environment [66]. Some species of fungi, including the *Boletaceae* family, exhibit below-average to high resistance to the toxic effects of hazardous elements. The available literature explains this issue in various ways, but the most scientifically supported are excretion of toxic metals, or reduction of uptake and efflux (efflux out) from cells [67], transport of toxic metal ions into cellular compartments [68] and complexation of metals with peptides, phytochelatins and metallothioneins [69]. Since the highest biological activity in the fruiting body is localized in the hymenophore, it is expected that the highest concentrations of this element will be localized there, which is also confirmed by our findings (Fig. 4).

The Cd content in the fruiting body ranged from ≤ LOD to 0.45 mg/kg. The sub-samples from lines A, B, J, K, L and M did not show Cd concentration above LOD (Fig. 4.). The Cd distribution was as follows: tubes and pores > flesh of cap > stipe.

Peptides, such as metallothioneins with chelating effects, which are found especially in the hymenophore, bind Cd in high concentrations [70]. This fact is probably the reason of our findings. Figure 4 shows that detectable concentrations of Cd were only recorded in the hymenophore and to a lesser extent in the part above the hymenophore, while the Cd concentrations in the stipe were below the detection limit.

### Lead (Pb)

Lead, unlike most other elements, has been shown to be actively excluded by edible mushrooms (BCF < 1) [71], making them relatively safe to consume from a lead poisoning standpoint. The authors measured Pb content in 109 samples across 28 wild mushroom species, finding that in nearly half the samples, Pb was more concentrated in the stipe than the cap. It is attributed to varying biological activity during the fructification influenced by protein content and the potential formation of metaloproteins [72]. Reported Pb levels in mushrooms range from 1.00 to 10.0 mg/kg [61], but most commonly average to less than 1.0 mg/kg [73]. Interestingly, Pb content varies considerably between species with different feeding strategies. Saprotrophic species tend to accumulate higher concentrations compared to mycorrhizal ones [71]. The Pb distribution within the mushroom itself also shows some patterns, with a tendency to be more accumulated in the stipe, although bioconcentration factors are similar for both major anatomical parts (depending on the species). An exception to this distribution pattern (applicable also for Co and Ni) are mushrooms from sites with significantly elevated Pb concentrations in the substrate [74].

Our results showed that Pb content ranged from ≤ LOD to 1.06 mg/kg. Figure 4 shows that Pb is accumulated strictly in the lower part of the stipe (lines I and J), with no detectable Pb content in the rest of the mushroom. The distribution of Pb was as follows: stipe > flesh of cap = tubes and pores.

### Nutritionally Nonessential Elements – NEEs

This group of elements includes metals and metalloids that currently have no defined biologically important functions. Of course, it is possible that they will be reclassified in the future as new knowledge is gained.

### Aluminum (Al)

Aluminum content in cultivated and wild mushrooms varies widely, from < 25.0 to 500 mg/kg [24]. Some authors reported Al concentrations above 500 mg/kg [22, 75]. The level of Al intake is largely dependent on the species, but there are also relatively large differences within the same species [76]. Bioconcentration characteristics for Al are insufficient, with BCF values below 1 for many species [77]. In exceptional cases, BCF values may reach the order of units [50, 76]. Mushrooms accumulate Al mainly in the caps, although this statement cannot be generalized [24].

**Fig. 5:**
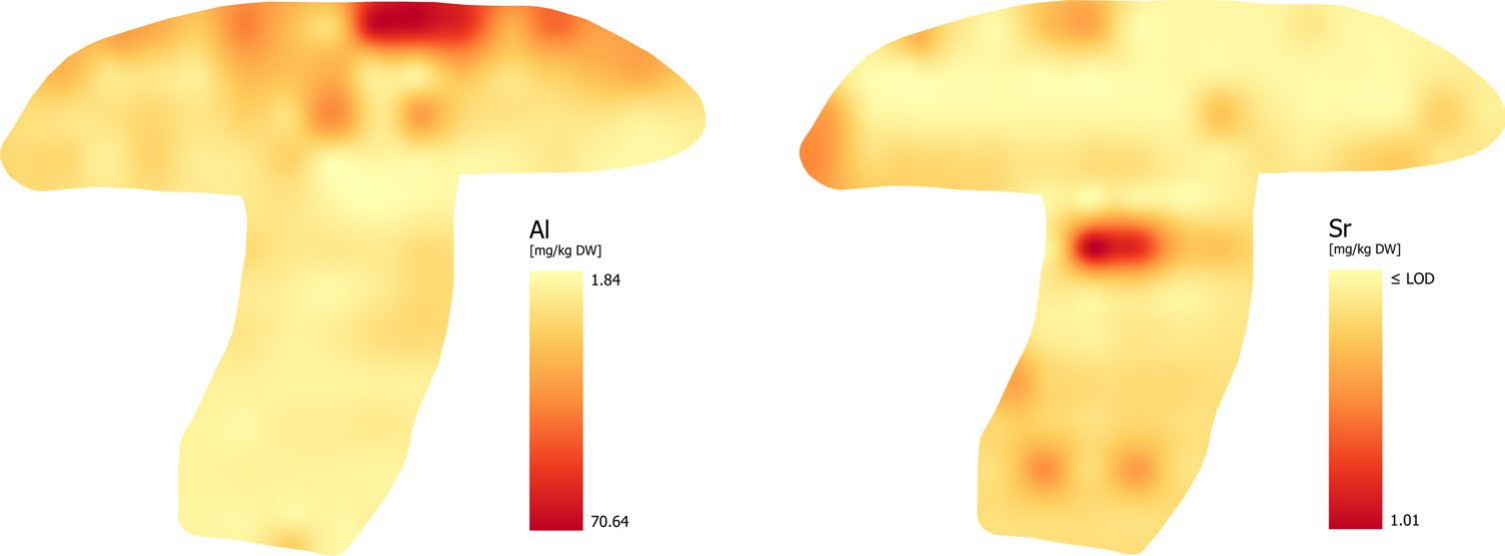
Distribution of Nutritionaly Nonssential Elements – NEEs (Al and Sr)

Our findings support the claim that Al is accumulated in the cap, mainly in the peel of cap and flesh of cap. The highest concentration of Al was found in lines A and B, where its content ranged from 8.58 to 70.6 mg/kg. The Al content in the stipe was significantly lower (< 4.00 mg/kg). Despite the clear concentration of Al in the cap as shown in the Fig. 5, the distribution of Al is unpredictable. The Al distribution was as follows: flesh of cap ≥ tubes and pores ≥ stipe.

### Strontium (Sr)

Based on available data the typical Sr concentration in wild and cultivated mushrooms, is below 2.00 mg/kg. The concentrations ranging from 2.00 to 5.00 mg/kg are also relatively common. Higher concentrations are typical for geochemically specific soils or contaminated soils [73, 78]. The distribution of Sr is generally uniform, but some publications point to small differences [33, 42]. Mushrooms take up Sr minimally and BCF values are generally less than 1 [42], which is also confirmed by radioisotope measurement of ^90^Sr [79].

Although the Sr distribution (Fig. 5) shows slightly higher concentrations in two specific locations (line F), the overall bioconcentration potential across the fruiting body is rather uniform and low (≤ LOD – 1.01 mg/kg) (flesh of cap = tubes and pores = stipe).

## Conclusion

The visualization method using QGIS software described in the manuscript represents a novel approach to the detailed mapping of elemental distribution in the mushroom fruiting body. The results indicate that this method can be a suitable alternative solution to the laser ablation coupled with ICP.

The findings highlight the importance of considering anatomical variations when assessing elemental content in mushrooms. The determined elements may be classified into the following three groups: *(i)* elements accumulated in the cap (Al, Ag, Ca, Cd, Cu, K, Fe, Mg, Ni and Zn), *(ii)* elements accumulated in the stipe (Ba, Mn, Na, Pb and Se) and *(iii)* elements characterized by non-specific accumulation (Cr and Sr).

The observed distribution differences suggest potential roles of particular elements in different parts of the fruiting body. High concentrations of the trace essential elements Cu and Zn and the major essential element Mg explicitly in the hymenophore may be linked to their role in the spore development. The distribution of the selected elements clearly points out that not only biogenic elements (Cu, Mg and Zn), but also elements with a detrimental effect (Ag and Cd) were accumulated in the hymenophore. One of the reasons is their biological equivalence i.e., the biological similarity of the elements in relation to their role in a certain process, regardless of the biological importance of the element (Zn – Cd).

Lead is one of the least mobile elements (especially in vascular plants). Mushrooms, except for some species, are generally considered to be Pb excluders. The results showed that a little amount of Pb was accumulated solely in the bottom part of the stipe. Based on the non-specific distribution of Cr and Sr, it can be assumed that their biological role in the fructification process is minimal, and/or insignificant.

The findings provide new insights into the mechanisms and patterns of elemental accumulation and distribution in mushrooms, contributing to our broader understanding of these processes. The study covers only one species and, as already mentioned, interspecific differences can be significant. Further research and assessment of the interspecies variability would provide better understanding of the elemental distribution.
